# "The Hospital" Nursing Mirror

**Published:** 1901-01-05

**Authors:** 


*The Hospital*, January 5, 1901.

### "The Hospital" Nursing Mirror.

Being the Nursing Section of "The Hospital."

[Contributions for this Section of "The Hospital" should be addressed to the Editor, "The Hospital" Nursing Mirror, 28 & 29 Southampton Street, Strand, London, W.C.]

### Notes on News from the Nursing World.

### THE QUEEN AND THE RED CROSS.

It is announced that the Queen has been graciously pleased to confer the Decoration of the Royal Red Cross upon Miss E. Bourguignon, Mrs. Bellingham, and Mrs. Drosthe, in recognition of the special services rendered to the sick and wounded, and to the troops generally, during the fighting in Tientsin in June to July, 1900. At the Watch Night meetings of the Red Cross Association in New York, on Monday night, the following message from Queen Victoria was read:—"I am deeply interested in the great work of humanity your society has undertaken, and send my best wishes for the new century to the American National Red Cross."

### THE APPEAL OF THE PRINCESS OF WALES.

The opening of the new year, and the new century, was marked by a characteristic appeal from the Princess of Wales, who never fails to turn to the best account an opportunity of doing her utmost to assist a deserving cause. As President of the Soldiers' and Sailors' Families Association she asks the public to come to the aid of the relatives of the rank and file, who are engaged in the war. The organisation in whose interest she pleads has been well supported up to now, and as much as three-quarters of a million sterling has been placed at its disposal. But it has 80,000 families to relieve, and as this means an expenditure of no less than £50,000 a month, its resources need to be replenished. We have not the slightest doubt that, in consequence of the Princess's touching appeal, the requisite help will be speedily forthcoming. The objects of the Association, the care of the wives and families of soldiers and sailors of the Queen, without any distinction and in whatsoever part of the world they may be serving, must recommend themselves to all sorts of people at all times, and especially while war is proceeding. Next week we propose to give some details of the nature of the work of the nursing branch, in which, of course, our readers are more directly interested.

### THE NURSE IN THE TWENTIETH CENTURY.

A series of articles on the "History and Development of Trained Nursing in the Nineteenth Century" were published in "The Hospital" Nursing Mirror of June 1899. There is nothing to add to them save that the war in South Africa has afforded the nurses of the present day a fresh opportunity of showing their courage and capacity, of which they have splendidly availed themselves. Practically, nurses were unknown even at the dawn of the century, though, thanks to Mrs. Elizabeth Fry, and still more to Miss Florence Nightingale, the situation was vastly changed long before its close. The trained nurse has made such amazing progress in the latter part that it would not be easy to set bounds to the triumphs she may achieve in the twentieth century. She enters it under fairer auspices than could have been considered possible even in 1860, when the Nightingale School was opened; and, with so many advantages which were denied to the pioneers of the profession, it will be her own fault if she does not steadily increase her influence, extend the scope of her ministrations, and make herself, as the decades roll away, a greater power—greater because of her increasing knowledge, her unfailing gentleness, her consistent unobtrusiveness, her womanly tact, her reckless devotion to duty, her unfailing belief in the nobility of her vocation.

### THE LIMITS OF A NURSE'S VOCATION.

The subscribers to the Maldon Nursing Association have been discussing a question which ought never to have arisen. It appears that a nurse in the employ of the Association refused to attend a typhoid case because she objected to the treatment adopted by the doctor. Instead of her action being condemned by the managers of the Association, they gave the nurse in question permission to refuse to attend patients under the care of the doctor whose treatment she does not deign to approve. Even if it had been alleged that the doctor was not qualified to discharge his duties, the course pursued by the nurse and her employers would have been open to criticism. But nothing of the kind is insinuated. The doctor, who is an assistant medical officer in the town, is well known and highly esteemed, and he admittedly has the confidence of the committee of the Nursing Association. The sole excuse of the latter is, in fact, that the nurse said she would prefer to resign rather than attend Mr. Price's cases; and because she has done good work they desire to appease her by the concession of a principle which ought not, under any circumstances, to be surrendered. Mr. Price, very naturally and very properly, determined to refer the matter to the subscribers, and at a meeting in Maldon he explained the situation. Nothing could have been more temperate than his statements. He said he had no desire that the nurse should be dismissed and he had made no complaints of her nursing; but he maintained that a nurse and a doctor each had their own province, and he could not consent to a nurse assuming his position. The ground he takes up is absolutely unassailable, and Dr. Reynolds Brown did well to strongly support him. Dr. Brown, in speaking of the nurse, said he should be very sorry to lose her, "but, if he found her criticising his treatment with any of his patients—whether she had a ticket from the Association or not—he could never allow her to attend any of his cases again." It is true that a member of the committee said he had, on its behalf, censured the nurse severely for having discussed the treatment with the patients, but what is the use of censure if you allow the person censured to go on having her own way? A nurse who sets herself up as a judge of the treatment of a case of the medical man under whom she is working exceeds the limits of her vocation and damages the profession to which she belongs.

### A BRISTOL SCANDAL.

We regret to learn that during Christmas this year the poor of a populous and necessitous district in Bristol—St. John's, Bedminster—were without a district nurse to minister to them. Owing to failure of subscriptions, the British District Nurses' Society had to withdraw her.

That this should happen in a large and wealthy city like Bristol is nothing short of a scandal. The Committee of the Society have always been in a state of anxiety as to the means of continuing it. Lately it has become acute, chiefly because of the death of many annual subscribers. But what are the living doing? Why do not they come forward, as they ought, to help an organisation of which the *Bristol Times and Mirror* says, "of the blessings of its labours to the people, there can be no doubt at all"? Our contemporary mentions two typical cases of patients of the Society who died last year. One was a Crimean veteran, who would undoubtedly have had to end his days in the workhouse but for the attentions which he received from the district nurse. The other was a private of the Gloucestershire Regiment, who was nursed through a long and tedious illness, his complaint being consumption. Surely, at a time when the very name of soldier excites enthusiasm, the Bristol people will not refuse to substantially support a society which cares for "old soldiers." It is not creditable that the recognition by Boards of Guardians and other public bodies of the work is little more than nominal; but it is disgraceful that private citizens should allow it to languish.

### LOCAL AUTHORITIES AND TRAINING.

The particulars of an appointment to the post of matron of Basingstoke Fever Isolation Hospital were courteously sent to us by the town clerk of Basingstoke, but as nothing was mentioned about the training of the selected candidate, in accordance with our invariable rule we requested the town clerk to supply the information. His answer was prompt, but not illuminating. All he could say was that "it does not appear, either from the application or from the testimonials, where Nurse Fletcher was trained." The obvious inference from this extraordinary statement would be that the new matron had not received a proper training. As a matter of fact, Miss Fletcher was trained at St. Mary's, and then at the Middlesex Hospital, where she was subsequently staff nurse from 1871 to 1874. Since 1877 she has been a member of the London Association of Nurses, and went to Basingstoke last July. Her credentials are, therefore, unimpeachable. But if local authorities do not take the trouble to make inquiries about the training of the nurses they engage, it is not strange that curious appointments are frequently made.

### A NOVEL DECORATION.

At the Yeovil District Hospital, where Christmas festivities were resumed last week after a lapse of some years, a novel feature of the decorations was a large spider's web made of wire, and surrounded with ivy, placed in one of the windows in the women's ward. In the centre was a large spider. The plants, decorations, and a great many of the presents for the patients, were provided by the matron, who also gave presents to the staff and the servants. The nurses, in their turn, presented the matron with a silver napkin ring, a tea-cloth, and a toilet set.

### THE BEER POISONING QUESTION.

An interesting contribution to the literature of beer poisoning is made by a hospital sister. She states that during the seven years she was in charge of a ward in a big London hospital, two or three cases of alcoholic neuritis were on an average admitted yearly to her particular ward. These were acute cases, lesser forms not being considered suitable for admission as in-patients. As the name implies, their condition was supposed to be due to the excessive use of alcohol, and the sister says that though the sufferers invariably denied this, and were even occasionally borne out by friends in their denials, "they were naturally not believed." Recent disclosures have, however, suggested to her that the personal habits of some of these patients "may have been misjudged." We agree with her as to the possibility, and may add that many other cases of beer poisoning in the past have probably been incorrectly described. But we are afraid that there are no means of rehabilitating those who have suffered from the misjudgment.

### LINCOLNSHIRE NURSES AT HAVERHOLME PRIORY.

A number of nurses belonging to the Lincolnshire Nursing Association have just been the guests of the President, Edith Countess of Winchilsea, at Haverholme Priory, Sleaford. This is not by any means the first occasion on which the nurses have been entertained by Lady Winchilsea, but unavoidable circumstances have prevented a similar gathering for the last two years. Twenty-one nurses arrived at Haverholme Priory on Saturday afternoon, and remained until the following Monday morning. On the evening of their arrival Lady Winchilsea addressed them on the nature of their work and its possibilities for good in its physical and moral aspects. She emphasised the great importance of cultivating tact, and said that the first step in acquiring it was to make a resolve never to repeat anything about a person but what was good. Alluding to the seven years' work of the Association, she traced its growth from the stage of infancy to what was now a sturdy childhood, but added that it had not yet reached the stature which she had in her mind when the Association was started. There were 20 districts in Lincolnshire in which a nurse was required. Sixty-two nurses had worked under the auspices of the Association since its formation. Of these 45 had been trained with the money provided by the County Council scholarships. Lady Winchilsea referred to the devoted services of Miss Glover, who now superintended the work, and also to those of Miss Sumpster, who had assisted so much at the start. After dinner a dramatic performance was given, the remainder of the evening being occupied with music and dancing. On Sunday morning a special sermon to the nurses was preached in Ewerby Church by Dean Young, and in the afternoon there was a service in the Haverholme Chapel. The guests thoroughly appreciated the kindness and hospitality of their President.

### MANCHESTER NURSING INSTITUTION.

At the thirty-fifth annual meeting of the Manchester and Salford Sick Poor and Private Nursing Institution, it was stated that the scheme for a new home for the Harpurhey district was meeting with most gratifying support. Mr. Mather, M.P., one of the speakers, also mentioned that "the great industrial classes are becoming more and more impressed with the character of the institution, and the immense effect for good it has on their domestic lives." He did not say how far their knowledge prompts them to contribute to its funds, which have been reduced to the extent of £40 on account of death and removals. Before the meeting closed a Roman Catholic priest complained that a Roman Catholic nurse belonging to the institution had been invited to join in prayers which were not in accordance with her religion, and that, consequently, several Roman Catholic ladies had refused to become nurses. It is a pity he added the arrogant assertion that "there was no other denomination in Manchester doing so much good work as the Catholics." But, of course, the religion of Roman Catholic, as well as of other, nurses, should always be respected; and any departure from that policy is inimical to the interests of the institution which permits it.

### A RED CROSS NURSE IN A CHURCH WINDOW.

In memory of one of the Imperial Yeomanry who died at Heilbron last September an extraordinary window has been erected in the parish church of Clacton-on-Sea. It represents, "in striking colour, the Saviour conversing with two groups of figures, among whom are a soldier in khaki, a Red Cross nurse, a man-of-war's-man, and a Zulu chief." This is called "a church window up to date." We prefer Church windows when they are not up to date.

### AN INCIDENT OF THE GALE.

Nurse Kelly, district nurse of St. Agnes, had last week a novel experience in connection with the wreck of the French barque, the *Seine*, off the Droslayn, Perranporth. News was brought into St. Agnes during the terrific gale on Friday morning that a large sailing vessel was in distress in the North Channel, driving with sails in ribbons in the tremendous seas, just out of the harbour. As it was uncertain where the vessel might be driven, the coastguards all along the shore were on the alert following her movements, till finally she struck to the west of the Chapel rock some little distance. But with infinite pains and tremendous exertions by officials and numerous residents of the district, all the crew were brought ashore. Meanwhile, Drs. Postlethwaite, of Perranzabuloe, and Whitworth, of St. Agnes, with Nurse Kelly, had been at work, and, assisted by the hotel proprietor and staff, had fires, hot baths, warm beds with plenty of blankets in readiness, so that the drenched and shivering French sailors were soon divested of soaked raiment and placed in a comfortable condition, and thereby probably saved from serious illness, which so often attends the survivors of wrecked vessels. Nurse Kelly's work at this little remote port seems specially varied. Not long ago the adventurers of West Kitty Mine resolved to contribute annually to the St. Agnes Nursing Association two guineas in recognition of services rendered by the nurse to the miners in cases of accident. And now comes the shipwreck incident at Perranporth, three miles from St. Agnes, in addition to ordinary district nursing. About six years ago the Redruth district nurse, Miss Brindly, had a similar experience, her services being utilised in connection with the shipwrecked mariners of the *Escurial* at Portreath, between three or four miles from her working centre. In this case one at least of the injured men required constant attention for a period of some weeks, pneumonia, with severe complications, following bitter exposure and buffeting of the waves; but with great care he finally recovered.

### NURSE AND NOURRICE.

The fact that nursing is no longer restricted in France to Sisters of Mercy has led to a difficulty about the name. There is a tendency to call the probationer at the training school lately opened in Paris *nourrice*, notwithstanding that she has no connection with wet-nursing, which necessarily results in confusion, as the following story shows:—An English lady, who prides herself upon her knowledge of French, lives in a garrison town in Brittany. Her eldest daughter is a nurse, and on her return from the London hospital where she was trained was introduced to a roomful of young officers by her proud mother as "My daughter who has been a *nourrice* in England." There was one moment of utter dismay, but the daughter being pretty and attractive, the officers rose to the occasion and realised the error.

### THREE PLUCKY NURSES.

It transpired at an inquest the other day on the body of a child who had died in the Scarborough hospital from exhaustion due to burns, that three of the nurses had permitted skin to be taken from them in the hope of saving the life of the child by grafting. The coroner highly commended the action of the nurses, and stated that they had voluntarily suffered much pain. We are quite sure that they do not regret their own suffering, though it was, unfortunately, borne in vain. These brave Scarborough nurses have afforded another illustration of the fact that it is easy to display courage without going to the front.

### PORTSMOUTH UNION INFIRMARY.

After a concert given last week for the benefit of the patients at the Union Infirmary, Portsmouth, and organised by the doctors and nurses and several local ladies and gentlemen, the following letter was presented to the medical superintendent by the patients:—"Dr. Knott, we all wish to thank you and Dr. Morley, and Uncle Taff, and the Chaplain and all our nurses who worked so hard to give us such a splendid entertainment. It helped to cheer us up a little so that we knew it was Christmas. Everyone of us was highly pleased, and very thankful to you all for thinking so much of us and taking so much trouble in our behalf. We hope they will always be happy and prosperous through life and may be spared to give so much pleasure to other people in years to come. We must not forget all the ladies and gentlemen outside, it was very kind of them to come from their homes to give pleasure to us poor people. John also we must not forget, we hope he will not work quite so hard at his Waxworks. We all remain, your humble servants, The Patients of B 1 Ward."

### SHORT ITEMS.

The patients of the Thompson Memorial Home, Lisburn, all of whom are incurable, have lately, by a sale of their work, been able to send a cheque for £25 to a sister institution, the Royal Victoria Hospital, Belfast.—The Board of Management of the East London Hospital are appealing for £8,500 to enable them to provide additional bedrooms and other accommodation for the nursing staff; to rebuild and equip the laundry and sundry domestic offices; and to add a new casualty department.—The Archbishop of Canterbury has promised to preside at the annual meeting of the After-Care Association for Poor Persons Discharged Recovered from Asylums for the Insane, to be held in the Library at Lambeth Palace on the afternoon of January 29th.—At St. Anne's, Soho, on Chrismas Day there was a special celebration of Holy Communion at 6 a.m. for the convenience of the many hospital nurses who attend that church.—The s.s. *Orotava* left Southampton on New Year's Eve, having on board Nursing Sisters F. M. Rennie and H. Hogarth, both of the Army Nursing Service. The s.s. *Avoca* arrived on the same date from South Africa. The following members of the Army Nursing Reserve were on board: Sisters Grewer, Lowe, Noble, McNeill, Leng, and Lamont; and Sister Makepeace of the Army Nursing Service. Sisters M. L. Pick and R. D. Cameron, of A.N.R., were also on board as invalids.—Mr. R. Kearton, F.Z.S., will deliver a popular lecture on "Wild Nature's Ways," showing birds, beasts, and insects living, loving, and labouring at home amidst their natural surroundings, illustrated by lime light views, to children at three o'clock on the afternoons of Jan. 10 and 11, in Steinway Hall. The profits are to be devoted to the National Orthopædic Hospital for the Relief of Poor Crippled Children.

### Lectures to Nurses on Medicine and Medical Nursing.

By NORMAN DALTON, M.D. London, F.R.C.P., Physician to King's College Hospital.

### I.—INTRODUCTORY.

THE chief duty of a nurse consists in her ministrations to the patient, such as the changing of the bedclothes, the cleansing of the body, the feeding, &c. These matters are, at present, admirably taught by the heads of the nursing profession in the institutions at which nurses are trained, but there remains a great deal of knowledge which a nurse should possess, which she can only learn from a physician.

In the first place, even in matters of pure nursing, it is sometimes necessary to depart from the routine methods of procedure. For instance, the feeding of a patient who is unconscious or of one who is suffering from paralysis of the mouth and throat is not only difficult, but is attended with danger, because small particles of food may pass into the windpipe instead of into the gullet, and even a drop of beef-tea may decompose in the bronchial tubes and set up a fatal inflammation of the lungs. This is one of the points which a nurse must be taught by a physician. As a matter of fact, it would be better to feed all such cases through a tube which has been passed safely into the gullet, but if the doctor does not think this advisable, the nurse can only remember the risk which the patient is running and take extra care in the feeding. In particular she must be careful to note that one mouthful of fluid has been swallowed before another is put into the mouth.

Secondly, in many diseases, very important symptoms may occur in the absence of the doctor, and it is essential that a nurse should be sufficiently well trained to observe these symptoms and report what has happened intelligently to the doctor. It is not, of course, necessary for her to understand the full significance of the symptoms, but merely to be able to give a good description of them. For instance, a child under treatment for bronchitis may also have whooping-cough, and as the characteristic cough may not take place while the doctor is present, he must rely on the nurse for the description of that cough. A more difficult and more important instance concerns the close observation of convulsions by a nurse. In many diseases of the nervous system and in some diseases (such as kidney disease) in which there is poisonous matter circulating in the blood, the patient may be seized with a convulsion or fit in the absence of the doctor; and, as his knowledge of the nature of the disease may depend on the exact movements which occur in the fit, it is important for a nurse to know what to look out for. For instance, if the convulsive movements occur on one side of the body (by which is meant the face, arm, and leg), the disease will probably be a tumour or some structural change situated in one side of the brain; but if the movements occur on both sides of the body, the disease may be true epilepsy or some affection produced by poison in the blood. There are many other important points to be observed during a fit, and these will be referred to under Diseases of the Nervous System. Where possible, a nurse should note down the movements as they occur, but, as a rule, she is too occupied with the patient, and is obliged to trust to her memory, which she should endeavour to train to accuracy.

Thirdly, a doctor has frequently to rely upon the nurse for a description of various materials which come from the patient, and which are the products of the disease from which he is suffering. Now a safe rule would be to preserve everything which comes from the patient for the doctor himself to see, and to let him know that this has been done, leaving him to decide whether he will look at it or not. This rule, however, might be carried too far; for instance, it would be absurd to keep the fæces for inspection in every case. Hence a nurse should have some idea of the materials which are important in different diseases, and preserve them unles told that it was unnecessary to do so. Particulars on this point will be given in these lectures. As regards the fæces, a small detail may be mentioned here, because if carried out it will earn a nurse the gratitude of her doctor, and that is, that she should always cover the bed-pan with a piece of glass before presenting the stools for inspection. It is not necessary to buy a regularly made glass cover, for an ordinary pane of glass does perfectly well, and if this cannot be packed in the small portmanteau of a nurse, it can be obtained anywhere in England, and the patient's friends will nearly always be willing to buy it for the occasion.

But there are some materials which become altered after they have left the body. For instance, blood may come from a patient in many diseases, either from the lungs, or the stomach, &c., and, if kept, it may be changed in appearance by becoming clotted before the doctor can see it. Hence a nurse should be trained to note and to describe correctly the appearance of the blood, whether dark or bright red, &c., at the time it was first seen. Again, perfectly healthy urine should be acid when it is first passed—i.e., if the blue litmus paper be dipped into it, the colour of the paper should become red. But after it has been passed it has a tendency to become alkaline—i.e., it will turn the red litmus paper blue. Sometimes in disease, or when large doses of certain drugs, such as acetate of potash, are being taken, the urine may be alkaline when passed from the bladder, and as it is important for the doctor, when he finds the urine alkaline, to know whether it has become alkaline or was alkaline when it was passed, it is necessary for a nurse to be taught the test with litmus paper.

Fourthly, in many diseases, certain symptoms are apt to occur unexpectedly, and to call for instant treatment. Now it is obviously unreasonable to expect a nurse to know the possible complications of every disease and how to deal with them; and it is the duty of the doctor to inform the nurse in each case as to what complications may arise. Still, as he cannot foresee everything, a certain amount of knowledge on the part of the nurse is of great use.

Lastly, not only should a nurse know which diseases are infectious and which not, but it is the bounden duty of the physician to keep her well posted up in the latest additions to our knowledge of the mode in which infection is carried in each disease. The importance of this knowledge is well illustrated by the case of typhoid fever. It has been known for very many years that the poison which causes typhoid is found in the stools, and that the disease is contracted by swallowing water, or milk, &c., which has been contaminated by the stools from a typhoid patient. Consequently there should be no risk in nursing a typhoid patient, provided that the stools, or the linen soiled by the stools, were immediately disinfected, and strict rules enjoining this disinfection exist in all hospitals. Nevertheless, nurses do appear to contract typhoid at times. Certainly they are more liable to it than doctors or students. Now this may be explained by the discovery in the last few years of the fact that not only are the stools of typhoid patients poisonous, but that living typhoid bacilli, i.e., the germs which cause the disease, exist in the urine. It is quite possible that, in the past, the elaborate precautions which have been taken as regards the immediate disinfection of the stools and everything touched by the stools has not been extended to the urine and the linen or utensils which are soiled by it; and there is reason to hope that the discovery of this additional source of danger will lead to a diminution in the number of cases, especially among nurses.

*(To be continued.)*

### Christmas in the Hospitals.

By Our Own Commissioners.

The festival of the last Christmas of the departed century was kept in the hospitals with all the brightness and the whole-heartedness which have always distinguished the sustained efforts made every year by those who minister to the sick and suffering to irradiate the anniversary with sunshine. In fact, each time the attempts to gladden the hearts of the patients by every conceivable means appear to be more ingenious and successful. If there was a period when the bare idea of spending Christmas in hospital seemed terrible, it has passed away. There remains, of course, the natural fear of pain, the dread of a long and trying illness; but, apart from this, thousands of toilers know that they are much more likely to spend a happy Christmas in the hospital than at home—so hard are the conditions of life to the multitude, so admirably have the authorities of the hospitals adapted themselves to the congenial task of introducing in their institutions the elements that render home attractive. The evidences of increasing, rather than of slackening, interest have been manifested during the past few days in divers directions. As of yore, the hospital staff has been well to the front. Bands of merry students have excited immense applause by their rattling entertainments; medical men of more mature years, and grave officials, have developed unsuspected powers of affording mirth; matrons have exhausted themselves, if not their resources, in planning programmes designed to give pleasure to everybody; and nurses, from the senior sister to the junior probationer, have worked unflaggingly in order to prevent a note of discord which might have disturbed the harmony—a hitch which might have proved disastrous. That all have gladly entered into the spirit of the thing is, of course, a truism; otherwise the actual labour of the Christmas season would have been a grievous burden. It is particularly pleasing to notice the increase of outside help. In several hospitals the musical features of the entertainments were mainly undertaken by friends of the staff: young ladies in charming frocks and young men in faultless attire had the satisfaction of feeling that they were contributing, in no small degree, to the enjoyment of the day. If the visitors who thronged the wards were not always discreet in their audible comments, many of them, by kindly smiles and well-chosen words of greeting or inquiry, showed a sympathy whose value may be measureless. In one institution, where a future Lord Mayor of London assisted to do the honours, a young Swedish gentleman, who had taken the opportunity of making himself acquainted for the first time with the interior of an English hospital at Christmas, expressed his keen delight with all that he had seen. While the enthusiasm of children culminated with the function of gathering the products of the Christmas tree, grown-up patients watched with eagerness the distribution of the endless variety of presents, and some of the oldest of the spectators became young again in witnessing the universal joy.

The new century will bring great changes, and it is impossible to say how the hospitals may be affected before its close, which none of us can expect to see. But at least one may be permitted to hope that the practice of keeping Christmas in hospital, by judiciously decorating, by the bestowal of inexpensive gifts, by the reasonable provision of good fare, by song and service, by all those little acts of graciousness and love that prove the presence in the world of the only kind of Christianity which is worth a single straw, will be maintained so long as there is human suffering capable of temporary alleviation, and it is possible to diminish, even for a fleeting hour, the sum of human unhappiness.

### Guy's Hospital.

Christmas Day began with the usual presents, and "stockings" for the children. After the patients' dinner, the "Disguysed Minstrels" went through the wards, beginning in Ruth (eye-wards) and ending in Astley-Cooper, giving a brief humorous entertainment in each. The wards were illuminated, and bright with decorations, and there were plenty of visitors. The great feature of the day was the tea in the out-patients' department, when over 500 children were entertained, and sent off with presents. On Saturday afternoon the annual treat was given in Martha Ward, consisting of Punch and Judy and a large Christmas tree. One of the doctors acted as Father Christmas. The tree and decorations were lighted with electric light.

### St. Thomas's.

Anyone who crossed Westminster Bridge in the dusk of Christmas Day would have known that something unusual was going on in the huge hospital by the river; for innumerable globes of light shone from the many windows—hundreds of Chinese lanterns hung in festoons across the wards. Within the patients were making merry with their friends; long churchwarden pipes had evidently been served out to the men, and the Blue Boracic Band was working very hard to entertain them while they smoked. The band is unique, and performed this Christmas for the second time. It is composed of nine of the students, who are got up as capital Pierrots, in white with large black pompons. The conductor of the troupe had thoughtfully provided himself with a pompon for each hand, as well as a small hat fastened on his head, in case his uniform sugar-loaf fell off, as it did in the course of his extremely energetic performance. At 4.30 the band triumphantly entered the Albert ward, having already given a performance an hour earlier in another ward. With strange, uncouth wind instruments and a drum, it marched, to the huge delight of patients, visitors, medical and nursing staff, and servants. Then followed an excellent programme of songs and instrumental selections, including (*a*) The Septic Serenade (S. Albus), (*b*) The Cyanide Symphony (Babinski), and (*c*) A Rhonchus in A flat (Gottsuchakoffski), under the conductorship of Signor Nicotini Nitch. After the National Anthem had been sung by everyone, tea was dispensed. "I never thought it would be like this," said a patient, lying in bed, as he took his cigarette from his lips. It was his first Christmas in hospital. The children's ward being closed, there was no tree this year. On the evening of Boxing Day, after the At Home given by the Nightingale Home, carols were sung by the probationers.

### St. Bartholomew's.

The wards at Bartholomew's were very effectively decorated, and there were Christmas trees in the Lucas, Elizabeth, and Stanley wards. Five hundred and forty patients took part in the festivities on Christmas Day, and everyone had plum pudding, as well as some special Christmas fare. Friends of the hospital gave presents, and the blue frocks of the children looked very bright and pretty in the wards.

### King's College.

The decorations at King's College Hospital were singularly effective, and the illuminations were on a considerable scale. On Christmas Day the patients were regaled with roast beef and plum pudding; and on Thursday, for several hours, the wards were visited in succession by a band of clever entertainers. Great credit is due to the resident medical staff and their friends for the ingenuity they manifested in the arrangement of the programme, and for the ability with which they carried it into execution. It would be invidious to mention names and impossible to give a full list, but the popularity of "A Village Blacksmith" was fully maintained by the possession of a very fine voice; a musical sketch, describing in laughter-provoking terms a remarkable trip from Charing Cross to Cairo, was warmly appreciated; and a performance, in character, by a supposed detachment of the police force received special marks of favour. Care was taken to prevent the patients in a serious condition from being in any way disturbed by the festivities, and though the fun was kept up with unabated vigour, it never became too uproarious. On Friday afternoon the children had their innings, and the nurses dismantled, for their benefit, the heavily-laden tree which made its welcome appearance in their ward on Christmas morning.

Charing Cross.

Christmas Day began for the patients at Charing Cross Hospital with the discovery of presents on the lockers, and very nice and useful presents they were; one patient who was eagerly watching for the promised visit of her "brother and his young lady" was rejoicing in the possession of four Christmas cards and a useful article of clothing, and saying how kind everyone was, and how different Christmas in the hospital was to anything she had ever imagined. The unusual smell of tobacco was in the men's wards, for the patients were allowed to smoke for once in the year. The sisters and nurses were hard at work during the afternoon decorating with evergreens, paper butterflies, and owls on sprays—wonderful birds of crinkled yellow paper with black beady eyes. Every cot in the children's ward had a butterfly and a bunch of violets, or something of the kind, fastened on it somewhere, and the little patients hugged their newly-acquired dolls and "golliwogs" with great glee. *Truth's* large doll was an object of great admiration: she looked on at the festivities with a consciousness that though children might come and go, she would go on for ever, for she is owned by the ward, not by any individual, as the matron was heard explaining to a small enquirer who "wondered who would have the big doll." During the afternoon the choir boys of St. Martin's Church sang carols, accompanied on the American organ by one of the nurses, and the sweet strains of "Noël, Noël" went through the open doors of the wards and into the drizzly outside air. The patients, who were allowed to receive an unlimited number of friends, had an early tea, and the tables were very prettily decorated with art muslins and plants. The decorations in all the wards were very pretty, and an immense amount of trouble was taken, though they were only to remain on the walls for one night. There was a Christmas tree on Thursday.

St. Mary's Hospital.

On Christmas morning every patient in St. Mary's Hospital received a present, thanks to the St. Mary's Aid Society and several Paddington ladies. In the children's medical and surgical wards a small tree, richly furnished, stood on the centre table and quite supplanted the old-fashioned stocking. The patients' dinner was served at twelve o'clock, the nurses dinner at half-past twelve, the dessert at the latter being given by Lord Rothschild. On Christmas Day and Boxing Day smoking was alllowed in the male wards. The decorations were simple, plenty of holly and ivy over the pictures and clocks and wherever it could be fixed up without nails, for no sound of a hammer was heard during all the preparations. In some wards, especially "New Boynton," the backs of the pianos were artistically draped with various shades and well-matched colours of crinkled paper. The "George Bird" ward was decorated entirely by the patients themselves. The walls were wreathed with paper chains of different colours, each little link was pasted into the next, every bit showed real work, and as no nails were allowed paste was used to fix them. Six large mottoes were also pasted up. The seven men were proud of their work: they sat round the fire on Christmas day puffing away at their pipes, and every visitor was told the whole plan of campaign. "We never troubled the Sister for anything but a pat o' paste. We did want our ward to be out of the common, them 'ere mottoes, specially 'Health to old friends and new,' are, to my moind fine, and Sister likes 'em and keeps a tellin' us how proud she 's of us all, bless her!" Between 4 and 4.30 p.m. the ward teas began, and visitors were expected to drink tea in every ward. In the Grafton ward there was a concert. Several ladies and gentlemen gave their valuable services, the programme including one recitation, four songs, and two violin solos, which the patients thoroughly enjoyed. In every ward something was going on, sisters, nurses, doctors, and students all worked hard to give a real hearty Christmas atmosphere to their surroundings, and to make up for the home longings of many of their patients, and right well they succeeded. When the time came to form a circle and sing "Should auld acquaintance be forgot" there was not a downcast face to be seen. All sang "God Save the Queen," and then the patients were put back to bed, the lights were lowered, and all was over again for another twelve months. The entertainment and Christmas tree for the children was held in the Board Room on Friday.

Westminster.

On Christmas morning, at Westminster Hospital, a beautiful Father Christmas went through the wards, clothed in his immortal scarlet gown with white cotton wool, and wearing a holly wreath on his brow and a necklace of bonbons round his neck. From his sack he brought forth presents for everyone, and merry jokes accompanied the giving of them. After the Christmas dinner an entertainment was given in the Board Room by friends, and the programme, daintily printed on cards, with the arms of the hospital, portcullis and crown in gold, included songs and recitations, a sketch, and conjuring. A large number of patients were present, some decked in coloured paper hats, which had an odd effect when worn on a bandaged head. The platform was tastefully decorated. In the wards, which were very pretty with evergreens, plants, and coloured lamps, there were bran pies, which admitted the display of much individual taste: one was like a large pink rose, and you "dipped" through a hole in the heart of the rose; another was a drum, and the third an enormous shoe. Sounds of merry laughter came from the "Incurables," who were having tea, and a group of carol singers were visiting the patients in another ward. The Children's Christmas tree was lighted by electric light.

London Temperance.

A large number of visitors accepted the invitation of the matron of the London Temperance Hospital to the Christmas Tree Entertainment on Friday evening. The gathering was held in a capacious empty ward, and the whole of the patients in the surgical ward with most of those in the others were well enough to come down and take part in the evening's festivities. The few who remained upstairs were not forgotten, the men being allowed to enjoy a smoke, while the women were otherwise consoled. An admirable and varied programme, including several plantation songs, two quartets, a trio, and two recitations, had been arranged, and was gone through with great spirit and success, Mr. Alderman Strong genially discharging the duties of chairman. Then came the excitement of the evening, when the brilliantly illuminated magnificent Christmas tree, whose branches reached to the ceiling and extended far into the room, was despoiled, and the wonderful assortment of presents was distributed. The articles on the tree were for the happy children, but there was also a big supplemental batch of parcels, and when these had been exhausted there was not a patient, nor a nurse, nor a servant, who had been overlooked. Thanks to the liberality of kind friends, the matron had been fortunate enough to collect a fund sufficient to provide a suitable gift for all. Throughout the proceedings the members of the staff were indefatigable in their efforts to promote the enjoyment of the patients, and prominent among the nurses was Sister Richardson, who, after serving in South Africa and suffering from an attack of typhoid, has now taken up her old duties with new pleasure.

HERBERT HOSPITAL, WOOLWICH.

For several days before Christmas at the Herbert Hospital the soldiers had been hard at work cutting reams of coloured paper into marvellous patterns, stringing evergreens for festoons, and painting badges and mottoes to deck the walls of their wards. The authorities were evidently determined to compensate as far as possible for the privations many of the men went through in South Africa a year ago, and the staff—military, medical, and nursing—laid themselves out for the pleasure and entertainment of those who were unable to spend Christmas with their friends, while all who could possibly go home were granted furlough. Tobacco was given freely, and on Christmas Eve the sister superintendent might have been seen counting the stores of oranges, apples, &c., and apportioning the good things for the morrow. The Christmas dinner was eaten in wards that surely never looked more gay and festive; for they were transformed by festoons of paper chains, many banners, various mottoes, and clouds of smoke from the pipe of peace. One poor fellow who has lost both legs was wheeling himself about on a chair, and looking forward to the time when he would be able to visit his English relatives, for he is a Canadian, and was at the front a year ago. He recalled sadly how many of his comrades were left dead on the field. It was quite pathetic to notice how "our comrades in South Africa" were remembered in the devices on the walls, and not less so to observe the simple reference to the widows and orphans of fallen comrades and the "In memory of our comrades who fell." The decorations in each ward were left entirely to the men, and it was whispered that a prize would be given for the best. "Sister" was requested not to give any "tips" to other wards, for each had some original and unique idea. In one it was the chalk blinds on the window panes, drawn by "the artist," in red studio jacket, and greatly admired by his comrades; here it was coloured paper curtains or doorway and window; there the chairs—huge armchairs in which to lounge comfortably—were entirely draped in the ubiquitous paper; one ward had hit upon the notion of decoration for the lockers, and another had shown a preference for its stoves, and the inability to find a dead bird to add to the realism of snow and withered twig was deplored by the orderly. Of course the portraits of the generals figured largely, surrounded by mottoes; and "Play up, Kitchener;" "Little man, big hero (Bobs);" "Tommy's Ideal (Buller)," mingled oddly with "Love your enemies" and "God is love." Nor were the Boer leaders forgotten; from a suspended umbrella hung the motto: "The thing to catch De Wet," while a classically minded orderly had constructed the following:—

"Oom Paul.
Da locum melioribus.
The Missing Link."

More than one ward had the pretty Irish words meaning "A thousand welcomes"—"Cead Milcfe Fadlthe;" and good wishes to the P.M.O., the doctors, nursing sisters, and visitors abounded; and in one corner, over the bed of a patient who had his family to see him, one read: "What is home without a mother?"

HOSPITAL FOR CONSUMPTION, BROMPTON.

The large entertainment room of the Hospital for Consumption at Brompton was filled from end to end on Thursday evening, when a splendid Christmas tree, lighted with electric light and growing all manner of fruit, was displayed on the platform. This was provided by Miss Heddy and the Lady Superintendent. The part of Father Christmas was humorously acted by one of the house physicians, clad in a gorgeous scarlet gown trimmed with cotton wool and sprigs of holly. He also wore grand patriarchal beard and flowing white locks. His opening speech was full of humour, and the recitation of an original poem, which, as he truly said, Tennyson himself could not have composed, gave great delight. The poem related the meeting, a year ago, between Christmas and his "old friend Joe," who shocked him by his appearance, wasted with disease. The patient comes into hospital, "upstairs he almost cut his way, his features were so keen." Then followed a description of the nursing staff.

The sister was a cheerful soul,
With manner kind and bright;
To make him easy and at home
It was her chief delight.

The nurses, too, a merry crew,
All day from nine to nine,
To make his lot a paradise
Did cheerfully combine.

And in the end the man is discharged, very tight in his clothes, and so heavy that the weighing machine breaks under him. The venerable Christmas then climbed the tall steps and threw down the first present from the tree, after which doctors, chaplain, and nurses distributed gifts in brown paper parcels, and crackers flew about the room. The scene was a bright and animated one, and patients, staff, and visitors enjoyed it thoroughly. "Sister, can't you get something for this side—the women are getting everything?" one heard at the bottom of the room; and Sister valiantly went to the rescue, and the shower of crackers and sweets came on again with fresh fury. Then a pianist played some cheerful dance music, and the curtain fell to an accompaniment of rapturous applause. Punch and Judy followed, and by the time the climax was reached the patients were quite ready for bed.

CANCER HOSPITAL, FULHAM ROAD.

Christmas was ushered in at the Cancer Hospital at 5.30 by the nurses and maids singing carols in the lower corridor. At 8 the matron visited the wards and distributed many useful presents. At one o'clock the patients enjoyed a good dinner. The wards were looking their best when the visitors arrived at 2.30 P.M. Each patient was allowed two friends to tea, but owing to the good nature of the nurses several had their little family around them. The ward tables were beautifully decorated, and there were plenty of evergreens throughout the hospital. The nurses worked hard, and waited on friends and patients with right-good will. After tea, when the fairy lamps and Chinese lanterns were lighted, the wards looked their best. At 5.30 the nurses and maids again sang carols, and a very pretty sight it was to see all grouped together in the corridor, and the staircase lined with the visitors and patients who were well enough to be up. The gong sounded at 7—a sign for all to depart—though the festivities did not altogether cease until some little time after the visitors had left.

### Samaritan Free Hospital.

On Christmas Day the patients at the Samaritan Free Hospital had a good Christmas dinner, or, as one of the nurses picturesquely put it, "a good tuck in"; then their friends came to tea, and afterwards carols were sung by the sisters and nurses. But Thursday was the great day, when a most successful entertainment was given by friends in the Nasmyth ward, and at 7 o'clock the presents, both useful and ornamental, were distributed. The matron takes a great deal of trouble over the selection of these gifts, and endeavours beforehand to find out what each one most needs. "I don't mind trouble at Christmas time," she said, "when it results in so much pleasure." Quite a number of patients were well enough to witness the entertainment, propped up with pillows; and six more were comfortably ensconced, two in a bed, along the wall; the servants had a good view of everything, and nurses and visitors (all friends of the institution) filled up the rest of the limited space. A convenient little stage had been erected in front of the large bay window; and with its piano, footlights, wreaths round the window, and suspended shield with XMAS above, it looked quite festive. Berried holly and laurel decked the pictures, overmantels, and gas chandeliers, from which hung graceful festoons. A large "Welcome" in red transparent paper was in the smaller window. The programme began with a piano solo, and then came a ventriloquial entertainment, which elicited great applause. A violin solo, an amusing farce, songs, and piano solos followed, and "God Save the Queen" wound up a most successful and pleasant evening, which everyone greatly enjoyed.

### Queen Charlotte's.

The decorations in the hall and on the corridors at Queen Charlotte's Hospital were "quite baronial," as the secretary said. In the vestibule was a holly bush that might have been transplanted from a country garden, and on the ornamental iron screen round the lift for carrying the patients up to the wards were very graceful trailings of evergreen. In the corridors festoons of green hung across from side to side. The holly, the gift of a friend of the hospital, was particularly rich in berries this year. No entertainments were arranged, but the nursing staff, and also the servants of the institution, are given tickets for the theatre.

### National Hospital for the Paralysed and Epileptic.

The wards, chapel, and other parts of the National Hospital for the Paralysed and Epileptic were decorated. On Christmas Day carols were sung in the early morning by the nurses. Cards and gifts were distributed at breakfast time, every patient receiving a suitable present. A service in the chapel, conducted by the chaplain, took place in the forenoon. A substantial dinner was provided, with dessert following. Carols were sung in the wards in the afternoon and evening. At the Convalescent Branch, Finchley, the Christmas Day arrangements were somewhat similar to those at the hospital. During the week an entertainment was provided, and a distribution of gifts from the Christmas tree was made. Ward teas took place in the several wards, and a special entertainment was given in the Duchess of Albany ward for children.

### Royal Hospital for Diseases of the Chest.

There are many hospitals with greater pretensions than the Royal Hospital for Diseases of the Chest in the City Road, but it would be very hard to find one where the patients more thoroughly enjoyed themselves on the great feast of Christendom, or where every soul in the establishment, whether secretary, medical officers, matron, sisters, nurses, or minor officials, combined to make it a day to be marked with a "white stone" by the eighty inmates. The Beatrice ward outshone the others in its display of holly, while in the other men's ward they were less lavish of greenery, but the fairy lamps were very effective. The women went in for more general adornment, and their ward looked very pretty. The centre of attraction in each ward was a Christmas tree, round which in the afternoon gathered the small fry, who by the generosity of the management accompanied their parents as visitors, and for four hours all was fun, frolic, and happy enjoyment, the tea and the concert which followed being equally appreciated. The visitors had departed by 7.30, and the patients were all soberly in bed by 8.30; but it wa slong before they were sound asleep, for, as a typical Bohemian occupying one of the beds observed, such a happy day "took a lot of forgetting."

### The Hospital for Sick Children.

Quite a crowd of visitors flowed into the Hospital for Sick Children on Friday afternoon, although the institution in Great Ormond Street had also been the scene of festivities on Thursday, when the out-patients were appropriately entertained. The whole of the expenses on that occasion were borne by the medical staff and their friends and the friends of the nursing staff, while the sister-in-charge of the out-patients organised the party. On leaving each little guest was presented by "Father Christmas" with a toy from the Christmas tree, chocolate, oranges, and a small parcel containing flannel clothing. On Friday the wards, bright with flowers and gay with innumerable fairy lights, presented a most charming appearance, enhanced by the Christmas tree which in each case formed the central attraction. Very noticeable was the unremitting attention paid by the nurses, clad in fresh pink gowns and white caps and aprons, to the little people for whose delight everyone catered. The majority of the children were able to enjoy their tea and to play on their bed table with the toys which were assigned to each of them. Many were so far convalescent that they quitted their cots entirely, and, under tender supervision, wandered about their ward listening quietly to the singing and playing of the ladies who gave their services, or gazing with wide-open eyes at the Christmas tree, bereft of its brilliant burden. Some, however, were not well enough to stir, and now and again one passed a little bed whose tired tenant lay fast asleep, its presents by its side untouched. One tiny mite was quietly slumbering with her arm round the neck of a still tinier doll. Here the recipient of toys lost no time in breaking the leg of a chair, or the arm of a wooden warrior; there the less destructive child was scrupulously careful not to damage its new possessions. Now and again a fitful cry was heard from a little sufferer, but the happy faces of the vast majority of inmates must have been an abundant reward to all who put themselves out of the way, and made any personal sacrifice in order to help the little patients to share the joy of the Children's Festival.

### The East London Hospital for Children.

Covent Garden had evidently been rifled by the Shadwell nurses, and somebody must have been very early to buy flowers for the wards in the East London Hospital for Children. Each of the bed tables had a dainty vase of violets or lilies of the valley; and on the centre tables were yellow daffodils, roses, and early anemones. Sacks of ivy—the closely clinging kind—had arrived from friends in the country, and most graceful tracery decked the walls of wards, passages, and out-patients' department. It was here that Friday's entertainment took place. After tea in the wards the little ones were carried down by nurses and gentlemen visitors, and laid on mattresses before Punch and Judy and two large Christmas trees. One patient was wheeled in, cot, charts, and all. Overhead were Chinese lanterns, and the Band of the Royal Horse Guards Blue played at intervals. A number of children from the Fire Station were also present, brought by their fathers and mothers, the former of whom watched over the trees with their many candles. Never, surely, was Punch taken more seriously than at Shadwell: "Did I throw the baby out of the window?" he screamed when Judy accused him of doing it on purpose. "Yes, you did!" twenty little voices answered without hesitation. When the little ones showed signs of getting tired the performance was wisely curtailed, with this undoubted advantage, that none of the coffin scenes came on. The children were all anxious to shake hands with "Daddy

Christmas" before getting their presents. It was touching to see one little girl, evidently a little mother at home, taking infinite care of the baby next her; indeed, a gentle spirit seemed to pervade the relations of the children with one another. The trees were the gift of a genuine supporter, and many friends sent toys, some of which, with a tree, are being sent to the convalescent home at Bognor.

EVELINA HOSPITAL FOR SICK CHILDREN.

The practice of inviting a crowd of visitors has been given up at the Evelina Hospital for Children, as it was thought that the excitement was not good for the children. And this year, on account of the closing of one or two wards, a public entertainment was quite impossible. The little ones, however, enjoyed their Christmas thoroughly: they woke to find presents awaiting them; they had chicken and pudding for dinner; "they sang, they danced, and they played games with the nurses, to their heart's content," said the matron, and no doubt they liked it just as well.

HOSPITAL FOR CHILDREN AND WOMEN, WATERLOO BRIDGE ROAD.

On Christmas Eve at the Hospital for Children and Women the children hung up their stockings, and Santa Claus did his duty by them as usual. The other patients received their presents in bran tubs, and the hospital was nicely decorated, though not in an elaborate style. The Christmas Tree Entertainment will take place on January 9th, from 3 to 6 p.m.

MARYLEBONE INFIRMARY.

The wards and corridors of Marylebone Infirmary were appropriately decorated for the Christmas season by the nurses, the officers, and the patients. Of the many novel designs none attracted more attention than a representation of Spion Kop. This was in one of the men's wards. It was erected on the floor, and mounted upon it were a gun and two cannon balls. Each of the latter weighed 14lb., and both were made by a blind patient out of odd scraps of tinfoil. On Christmas Day the patients were treated to the usual good fare of the season; and the children, of whom there are more than a hundred, were each presented with toys.

BEDFORD COUNTY HOSPITAL.

Christmas festivities have been celebrated with great success at the Bedford County Hospital. The patients seem to have enjoyed the various entertainments provided for them, and appreciated their presents very much. On Christmas morning the choir boys sang carols in all the wards, dinner at 12, and in the afternoon the chairman and matron went round the wards giving presents to each patient. As there is no Christmas tree this year the children had stockings hung on their cots, and were awake *very* early in the morning to see what Santa Claus had brought them. In the evening a party of ladies and gentlemen played and sang in all the wards, the men thoroughly enjoying the concert, and the smoking which was allowed on that occasion On Thursday afternoon the entertainment was in the women's block, beginning with a little play acted by four of ths nurses, which was very amusing and bright. Then songs, recitations, violin solos, &c., by the sisters, nurses, and friends brought by one of the surgeons who was responsible for the evening's amusement. The festivities ended on Friday by another concert in the men's block, arranged by some friends who brought a party, and which was also very successful.

ADELAIDE HOSPITAL, DUBLIN.

A very pleasant entertainment was that given on New Year's Eve by the superintendent and sisters of the Adelaide Hospital, Dublin, to a number of the institution's friends. The invitations were from four to six. May's excellent band was stationed in the hall, and played all the afternoon, to the great enjoyment of patients as well as visitors. The fine staircase, which is such a decorative feature of the "Adelaide," was wreathed with evergreens, and hung with fairy-lamps and Chinese lanterns. Perhaps the (human) decoration of pretty nurses in smart navy uniforms and the crispest of caps and aprons was even more widely admired. Tea was served in the children's medical ward, cleared for the occasion, and adorned with evergreens and fairy-lamps. The large crowd of guests were ably attended to by Miss Fitzpatrick and the sisters, and enjoyed themselves greatly. One of the chief attractions of the afternoon was the appearance of the famous soldier boy, "Bugler Dunne," who is quartered with the Fusiliers in Dublin at present. Everyone was anxious to speak to him and shake hands with him. The little hero received his honours with perfect simplicity and dignity, being apparently quite unspoiled by all the adulation he has received. The superintendent and sisters are to be congratulated upon a very successful afternoon.

PORTSMOUTH MILITARY HOSPITAL.

Christmas passed off in a very cheery manner at the Military Hospital, Portsmouth. Most of the patients being convalescent were able to enjoy their dinner of roast beef and plum pudding, and to do full justice to the bountiful tea provided for them. This latter meal was held in an empty ward beautifully decorated with cut flowers, ferns, evergreens, and scarlet draperies. The sisters—with the help of some ladies and officers—waited on the men, of whom there were about eighty. A smoking concert and an exhibition of conjuring followed, and closed what had proved to be a pleasant day for the sick of the garrison.

NORWOOD COTTAGE HOSPITAL.

Christmas was a most enjoyable time at Norwood Cottage Hospital. It began as early as 4 A.M., when some of the patients discovered that during the night "Santa Claus" had paid them a flying visit, leaving behind him a legacy in the shape of a "big stocking," *i.e.*, a pillow-case stuffed full of goods things. The quiet wards soon become like a fair, beds being covered with toys, chocolates, &c. There were also plenty of useful presents, articles of warm clothing, &c., sent by the many friends of the hospital. One of the medical staff sent in a new half-crown for each adult patient, and a new shilling for each child. Another doctor gave the matron a sovereign to buy things for them. At mid-day a good dinner was provided for the patients, and most tastefully laid out in the pretty day-room by the matron. In the afternoon there was a patients' "At Home," and each one had a few friends to tea, and spend the evening. In the evening they had a bran dip. They also had music and singing, and there were games of "hunt-the-slipper" and "musical chairs," in which the matron and nurses joined to the great delight of the patients.

ANCOATS HOSPITAL.

The patients at Ancoats Hospital, Manchester, received presents of shawls, petticoats, shirts, and socks, and a Christmas tree was provided for the children's ward. On Christmas Day the patients were treated to an excellent repast. The Ladies' Committee gave a contribution of books to the nurses' library for their Christmas gift.

WEST CORNWALL MINERS IN HOSPITAL.

Patients of the Miners' Hospitals at Redruth had a very enjoyable time. Pretty decorations of holly, red and variegated laurels and greenery, Chinese lanterns, and looped drapery added to the festive appearance. In the children's ward on Christmas morning the youngsters found gifts in the shape of toys on their cots and little beds, and all the other inmates at breakfast had parcels containing pretty and useful presents of warm clothing, &c. The men in the accident ward were given special permission to smoke. Contributions toward presents had been received by the matron from the medical staff, members of committees, and other friends. Christmas fare was amply provided. A Christmas tree for the children laden with toys was a source of delight to all in the evening. Everything possible was done by the matron and staff to add to the general enjoyment, and at the close of the proceedings a deputation of patients expressed the thanks of the inmates to all who had helped in giving them such a pleasant Christmas.

### Queen Victoria's Jubilee Institute for Nurses.

HER MAJESTY has been pleased to approve the appointment of the following to be "Queen's Nurses," the appointment to date January 1, 1901:—

ENGLAND.—Violet Charlesworth, Jeanie Akeroyd, Alice Mary Gibbs, Rosita Macandrew, Mary Eva Beryl Campbell, Lilian Holland Bullock, Jane Tyson, and Emma E. Chamberlain, London; Annie L. Lenton, Ruth Shimmin, Laura Perry, and Jane Haydon, Liverpool; Agnes Crossley, Mary Agnes Hurley, Edith Martin, and Mary Readding, Manchester; Mary Ellen Larkin and Maud M. Phillipson, Salford; Fanny Sarell, Birmingham; Annie Bulton, Bilston; Fanny Noise, Chorlton-cum-Hardy; Jane F. D. Ross and Margaret Wilson, Sunderland; Lizzie Osborn, Carey Nichol, Henrietta Le Mare, Eva A. Sorsby, and Martha Barnes, Leeds; Emily Lambert, Gateshead-on-Tyne; Belle Sutcliffe, Dorothy Godden, and Edith M. Buller, Brighton; Marian Ellen Earp, Warrington; Susanna M. F. Bylee and Frances Madeline Lang, Huddersfield; Mary Smith, Lancaster; Annie Clay and Eliza Jane Fews, Bath; Amy F. Cochrane and Nellie Ashley, Crook; Ann H. Smith, Cockermouth; Irene B. Smith, Coxley Green; Mary H. Robinson, Cleator; Emma Gladwin, Colchester; Jessie Mackay, Cleckheaton; Adelaide J. Pringle and Jane A. Higham, Bramley; Minnie Bishop, Hadleigh; Jessie Jeffery, Doncaster; Ethel M. Bristow, Grantham; Helen Clarke and Frances Cook, Grimsby; Agnes Leach, Haddenham; Georgina M. Green, Minchinhampton; Alice M. Watts, Melbourne; Beatrice E. Olphert, Norton; Anna M. Wisdom, Reading; Agnes McGregor, Rochdale; Alice A. Nichol and Mary E. Mombert, Stafford; Mary J. Roberts, Ulverston; Clara Essex and Sophie H. P. Sulivan, Windsor; Martha White, Alnwick.

WALES.—Mary Thomas and Alice Walmsley, Bangor; Elizabeth G. Grigg, Carnarvon; Caroline A. Lawson, Pontypridd; Edith Alice Rose, Kilgerran; Kate A. Williams, Llanerchymedd; Mary Price, Towyn.

SCOTLAND.—Sophia Cowan Macrae, Edinburgh; Isabella Austin, Jane McBryde, Mary McEwan, Ann Findlay Sim, Robina Walls, and Jane Baird, Glasgow Eugenie K. Irving, Dundee; Marian N. Anderson, Paisley; Menella Knight, Blairgowrie; Agnes Jane Ormiston, Wick; Marjorie Fraser, Lamington; Marion B. Law, Newton Stewart; Kate W. Jones, Blantyre; Annie McColl, Ardrishaig, Argyllshire; Annie B. Smith, Craignish, Argyllshire; Helen Nicolson, Tarbert, Argyllshire; Kate McKinven, Strachur, Argyllshire.

IRELAND.—Emma Walker, Frances E. Stammers, Jane Rogers, Charlotte Parke, Elizabeth Heaps, Gertrude Bee, Dublin; Josephine C. Higginson, Londonderry; Elizabeth Annie Stubbs, Bushmills.

### The New Offices of the Royal British Nurses' Association.

THE offices of the British Nurses' Association, of which Princess Christian is President, have been removed to new premises at 10 Orchard Street, W., where all communications for the future should be addressed.

The new offices are much more commodious than those formerly occupied by the Association, and the advantage of having the entire house is obvious. There is a large double office, fitted with Stone's patent sliding doors, which can be thrown open for meetings and which will seat about a hundred people. All the business meetings of the Association will be held here, as well as the lectures. The room is well lighted at both ends, airy, and quiet. The Nurses' club room and tea room are on the floor above. The office of the Auxiliary Society is in the same building, which it is hoped will prove a useful arrangement, as although the Auxiliary is financially quite separate from the Association, it is engaged in carrying out one of the principles of the Charter, in providing a bureau where nurses may be procured at short notice. Some confusion appears to exist in the lay mind on this point; and letters intended for the Auxiliary are frequently sent to the Secretary of the Association; much delay may therefore be saved by having the offices under one roof. The new premises will be open for inspection on January 17th, when there will be tea and coffee at 4 o'clock, and at 5.30 Mrs. Humphry Ward will open a discussion on the education of crippled children, in which the matrons of various Children's Hospitals will take part. This will be considered the "house-warming" of the new premises, although official work has not been suspended by the move. Tickets for the 17th may be obtained from the Secretary.

### The Nurses' Bookshelf.

ADVICE ON ACCOUCHEMENT AND THE MANAGEMENT OF BABIES. By "Sister Marian."

THE advertiser is an interesting personage who crops up in all sorts of unexpected quarters. This is a sweet little book, in elegant paper cover, tied up with ribbon—and in a bow, too!—and it contains a great deal of information sure to be of much utility to young married ladies looking forward to that great event, their first accouchement. It is very well done, and everything is very nicely put; but, after all, the true inwardness of its publication is to advertise Hartmann's Wood Wool, his sheets, his towelettes, and all his other arrangements for ladies at that interesting period when babies form the centre of their thoughts. However, if we are to have advertising let us have it in neat artistic form like this; and although no doubt this little pamphlet will do good to Mr. Hartmann, it will do no harm to those who read it, for a young married woman might do far worse than get her mind thoroughly instilled with the good, clean sanitary notions which these Wood Wool preparations have done so much to popularise and to put in practicable form.

### Death in our Ranks.

THE happy Christmas which was being spent in the Royal Infirmary, Windsor, was suddenly marred by the unexpected news of the sad and untimely death of one of the most esteemed nurses. A correspondent writes: "Nurse E. C. Walker had just completed her three years' training in the Infirmary and received her certificate on November 1st last. She left to enjoy a well-earned holiday before taking up work afresh, exactly a month before the day of her death, on December 29th, in her usual bright spirits, full of life and promise, taking with her the good wishes of the staff and patients with whom she had worked so willingly for the past three years. Her kind manner and general brightness endeared her to all, and her willingness to spend and be spent for others, both when 'off duty' as well as 'on duty,' made her a universal favourite."

### Minor Appointments.

ROYAL EYE HOSPITAL, MANCHESTER.—Miss Maud Mason has been appointed sister. She was trained at St. Bartholomew's Hospital, Rochester, and has been charge nurse at the Brook Hospital, Shooter's Hill. Since then she has been engaged in private nursing in London.

HASTINGS UNION.—Miss Eliza Cage has been appointed assistant nurse. She was trained by the Meath Workhouse Nursing Association at the Home for Confirmed Invalids, Aubert Park, Highbury.

### Echoes from the Outside World.

AN OPEN LETTER TO A HOSPITAL NURSE.

My first letter written to you in a new century must, of course, begin with what is called the "Compliments of the Season," but I do not know if it would be more correct to give any special greeting on such an occasion, because it is my first experience of so marked a transition from one epoch to another. I am not like Mrs. Betsy Moore, of Bishops-Teignton, Devon, who enjoys the rare distinction of having lived in three centuries, though I do not fancy she can have had a very vivid recollection of the first of the trio. I cannot, however, go far wrong if I hope that the year to come may be laden with many blessings for us all, foremost amongst these the blessing of peace, which at present, alas! seems almost as far off as if Lord Roberts had not returned to England. The finish of the Nineteenth Century has been a sad one to many of us; even the very end, almost the last day, brought ruin to several well-known firms on the Stock Exchange, who a week before had no idea of disaster. The First of January was ushered in by a religious service at midnight in a large number of churches and chapels. Even the clergy who usually object to the sensationalism of these services, recognised the uniqueness of the present occasion, and St. Paul's Cathedral had a service at seven o'clock on December 31st—a distinct innovation. Long after the last worshipper had left the sacred edifice the streets were full of men and women waiting to hear the chimes ring out the last stroke of the hundred years. The New Year Honours came as a surprise to those who thought the war distinctions would have been published. These are evidently left till later, and meanwhile the only fact of much interest to nurses is that a baronetcy is bestowed on Drs. Church and Barlow and a knighthood on Dr. Hugh Adcock, whilst Mr. Cooper, of the Gables Hospital, Surbiton, who has been so generous in receiving wounded soldiers and caring for them, becomes Sir Alfred.

Christmas Day for those who are no longer young very often partakes as much of sadness as of mirth, and for the Queen the festival this year must have been greatly overshadowed by the death of the Dowager Lady Churchill. For forty-six years "Jane," or "Jane Churchill" as her Majesty frequently calls her in the royal books of memoirs, has been not only lady-in-waiting, but also the intimate and constant companion of the Queen, and the shock of hearing that she had been suddenly called away, found dead in her bed on Christmas morning, must have much distressed the Sovereign, whose friends are necessarily limited in number, and perhaps, in consequence, more appreciated. Lady Churchill, it is commonly said, knew all the secrets of the Royal household, because the Queen had found by experience that anything whispered into "Jane's" ear was as safe as if the communication had never been made. Though seventy-four years of age, Lady Churchill looked comparatively young, and carried herself well, but she was not as strong as her royal mistress in some ways, for two or three years ago she was obliged to give up accompanying the Queen abroad in the spring owing to feeble health. At her funeral on Saturday, when the Royal yacht fetched the coffin from Osborne, by the special desire of the Royal mourner, a large number of the Royal Household attended, and wreaths from the Queen and other members of the Royal Family were laid upon the coffin.

Although Lord Armstrong had not long retired from active life, the fact of his being still alive may possibly have been unknown to some of you. The celebrated engineer had passed the age of 90, and it was half a century ago when his name, as plain Mr. Armstrong, was in everyone's month. He turned his attention to improving the kind of gun used by the English artillery, which had practically made no advance since the Peninsular War. The great difficulty was how to construct a weapon of which the walls would stand an explosion strong enough to materially increase the velocity of the projectile. This puzzle, by means of a steel core and other clever arrangements, he solved, and so produced an aid to warfare much more terrible and dependable than any the world had ever seen before. An English committee appointed to enquire into the merits of the gun decided in favour of using it at once, and Mr. Armstrong was free to put his own price on the invention. Instead of doing so he presented his patents to the nation. Then a strange thing happened. After paying in four years for guns made in the factory over a million pounds to the Elswick firm, of which Mr. Armstrong was the head, Lord Palmerston's Government stopped all orders, and paid £65,000 for the privilege of breaking a contract by which Elswick was to supply the Armstrong gun to the English Government and to no other country. This was in 1863, and some changes have been made since, but practically with a few improvements as to slow burning powder and other details the gun perfected by Lord Armstrong fifty years ago remains still in use now that the great man has been gathered to his fathers, and his name as an inventor gone down to posterity.

I have just received a letter from a lady in Hong Kong. We often growl at the English climate, and its sudden and wonderful changes, but on the whole it seems distinctly better than some other places. You may remember that the fact of a typhoon having visited Hong Kong was telegraphed over some little time since, but this is the first mail which has reached us since the disaster. My correspondent says: "Our servants' quarters were entirely blown down, and they only just escaped with their lives. It took a few moments to recover from the paralysing effects of the terror inspired by the fearful noise of the wind, which was so great that it prevented anything being heard but itself. We felt as if we were the only people left in the world, for not a sound could we hear from our neighbours, although we live in a terrace, and many of them were undergoing similar experiences to our own, hammering up doors, and barricading half the night. As the servants did not come in to us, and we had heard nothing of them, I went into the kitchen, and was horrified at the sight which met my eyes. The terrified domestics were all huddled together in a corner of half the kitchen. The rest of the building had disappeared, leaving nothing but a mess of bricks and mortar behind. The chimney no longer existed, and though a fortnight has elapsed since the accident, not a single bricklayer will come near us; even the offer of double pay produces no result, so much damage has been done. The gale of wind will cost us at least 1,000 dollars, besides untold discomfort. The servants have to sleep in the basement, or wherever they can manage a shake-down, as their proper apartments have gone; every scrap of food we eat is smoked fearfully, because there is nothing to carry the smoke away; the servants, naturally enough, grumble at all they have to put up with, I do the same; and as to the garden, it is simply wrecked. The peas were in pod, the beans flowering, and the turnips forming. The plants were blown to pieces or torn up by the roots, whilst the flower seedlings were not to be found. Such wholesale destruction nearly broke my heart, and the visitation seemed all the worse because we always consider that we are out of the wood after October. I can never remember a typhoon occurring in November, and everyone admits that it is the most violent we have had since 1894. Assuredly, the last year of the nineteenth century has not been kindly disposed to English residents in China!"

### Examination Questions for Nurses.

### EMERGENCY NURSING.

### Result of December Competition.

The question was as follows:—"How would you prepare a bed for a case of fractured thigh at half an hour's notice in a house belonging to a middle-class patient?"

### The First Prize.

Nurse Lucy takes the first prize. She is evidently a person of many resources, and understands how to make the most of materials at hand without sighing for those which are not. In private nursing resourcefulness is a most valuable quality: it is so frequently necessary to use not what one *would*, but what one *can*, and an inventive mind in emergency nursing is often far more useful than deep professional learning.

I do not quite know why Nurse Lucy wishes to deprive the sufferer of his pillow. It is well to keep children quite flat, but a pillow may always be allowed to an adult, and in the case of aged or very stout people it is often necessary to raise them considerably at the shoulders, lest whilst you cure the fractured thigh your patient slips through your fingers from pneumonia, brought on by lying on the back entirely. This is a very serious danger with old people. In advanced age there is very generally insufficient union of the fracture, so that absolute stillness and a perfectly good position are not of primary importance, whilst the danger of shock, alteration of long-continued habits, and the rapid development of pneumonia are evils that should be present in the nurse's mind.

### The Second Prize.

Nurse Nellie takes the second prize. She also has practical ideas and knows how to make her surroundings fit in to her needs.

### Honourable Mention.

Eilrah, Nurse Esther, and Nurse Hutchinson gain honourable mention. Eilrah's paper is very good: she must be congratulated upon being often among the successful competitors. Several candidates make one serious fault. They speak of the necessity of a mackintosh, but they place it *under* the bottom sheet, where it is practically useless. The mackintosh must be across the bed between the bottom sheet and draw-sheet, so as to obviate the danger of the under sheet first becoming soiled, and then the patient being unnecessarily moved whilst it is changed. In what way would matters be improved by a mackintosh underneath?

Another error is to put a blanket between the under sheet and mattress. There is no escape for perspiration, and the blanket in a short time becomes damp, and has a most injurious tendency in the direction of bed sores.

### Answers to Question for December, 1900.

### First Prize.

I should hope to find an ordinary wool-mattress, and *not* a feather bed, and should then place boards underneath the mattress to make it firm and straight. If suitable boards are not available a large paste-board and one or two framed pictures of equal size can be used, turned face downwards of course.

Next to the undersheet, and under the draw-sheet, a mackintosh is a necessary precaution; but failing that a thick double draw-sheet will do. No pillow should be used. A cradle, to keep the weight of the bedclothes off the patient, can be made from a piece of wire-netting, arched, and two stakes or thin bamboo sticks passed lengthwise in and out of the holes, with a crosspiece in the shape of the letter X at the foot to keep it firm and allow room for the extensions. I should have two blocks of wood ready to be placed under the casters to raise the foot of the bed. Weights borrowed from the kitchen scales would be useful, according to the weight of extension ordered by the doctor.

Nurse Lucy.

### Second Prize.

A horse-hair mattress is obtainable in most houses, but failing this a straw mattress, though very hard, would do.

Between the mattress and the bedstead one or two long strong pieces of wood must be placed (ironing boards would do nicely) to prevent the mattress sinking in the middle.

Over the bottom sheet a draw-sheet and mackintosh must be placed For the former an ordinary sheet folded lengthways would do, and if a mackintosh is not to be had a piece of oilcloth might do temporarily, providing it was waterproof.

The bed should be warmed by hot-water bottles or other means; and to elevate it at the foot, if no blocks of wood are available, two bricks could be hollowed out a little (with a chisel) and placed under each foot of the bed.

To prevent the weight of the bedclothes on the injured limb a backless chair might be used if the lower staves were taken out and the legs shortened to a convenient height, or a basket or bandbox might if cut be utilised as a cradle.

Nurse Nellie.

### The Question for January.

In the case of a man severely kicked in the abdomen by a horse what are the chief dangers to be apprehended, what symptoms would you expect, and what course of treatment should you pursue, supposing medical assistance not to be at once obtainable?

#### Rules.

The competition is open to all. Answers must not exceed 500 words, and be written on one side of the paper only. The pseudonym, as well as the proper name and address, must be written on the *same* paper, and *not on a separate sheet*. Papers may be sent in for fifteen days only from the day of the publication of the question. All illustrations strictly prohibited. Failure to comply with these rules will disqualify the candidate for competition. Prizes will be awarded for the two best answers. Papers to be sent to "The Editor," with "Examination" written on the left-hand corner of the envelope.

N.B.—The decision of the examiners is final, and no correspondence on the subject can be entertained.

In addition to two prizes honourable mention cards will be awarded to those who have sent in exceptionally good papers.

### Appointments.

Royal Victoria Hospital, Belfast.—Miss Mary Hawkins has been appointed sister. She was trained at the Royal Cornwall Infirmary, Truro, and the North Staffordshire Infirmary, Stoke-on-Trent, and has since been charge nurse.

Officers' Hospital, Murree, Punjab, India.—Miss A. J. Weighell has been appointed by Lady Roberts lady superintendent. She was trained at the Royal Infirmary, Bristol, where she was subsequently staff nurse. Since November 1890 she has been engaged in nursing in the Indian Military Hospital.

Sudbury Union Workhouse.—Mrs. Nelson, *née* Wimberley, has been appointed matron. She was trained at Mile End and St. Pancras Infirmaries, and has been charge nurse at the latter institution, also superintendent of nurses at the Bethnal Green Infirmary. Her last appointment was that of matron of the Romney Marsh Union Workhouse which she held for three years and nine months.

Borough Sanatorium, St. Helens.—Miss Margaret Burgess has been appointed matron. Miss Burgess was trained at the Adelaide Hospital, Dublin. Her subsequent appointments have been charge nurse, Charlemont Street Private Hospital; night superintendent, Adelaide Hospital; assistant matron, Cork Street Fever Hospital, Dublin; and matron of the County Meath Infirmary for nearly five years.

### "The Hospital" Convalescent Fund.

The hon. secretary acknowledges with grateful thanks the receipt of 5s. from Miss C. E. Gardner; 1s. 6d. from Nurse E. F. Roche Smith; 5s. from Miss E. M. Williams; 3s. from Nurse E. M. Geach.

### Presentations.

LEICESTER INSTITUTION OF TRAINED NURSES.—The nurses of the Institution of Trained Nurses at Leicester presented the Lady Superintendent, Miss McHardy, with a Queen Anne silver tea service on Christmas Day as a small recognition of her kindness and consideration to them during the past year. Miss McHardy gave the nurses a dinner in the evening, and all who were able to partake of her hospitality were present.

SKELTON MINERS' ACCIDENT HOSPITAL.—Miss Sweet, who for five and a-half years was district nurse and midwife, and subsequently for four years nurse-matron at the Miners' Accident Hospital, Skelton, Cleveland, has been presented with a travelling clock and a purse of gold. She left in September to take up district work again, and the presentation was a pleasant surprise on Christmas Day.

HORSHAM UNION INFIRMARY.—On resigning her appointment as head nurse at the Horsham Union Infirmary, which she has held for nearly six years, Miss Alice Proudfoot was presented by the patients, chaplain, and nurses with a beautifully fitted dressing case, and by the lady visitors of the Infirmary with a silver afternoon tea service.

BLACKHEATH AND RICHMOND NURSING INSTITUTIONS.—Miss Duncan, superintendent of the Blackheath and Richmond Nursing Institutions, was presented at Christmas with a Queen Anne cream jug and sugar basin by her staff.

ROYAL EDINBURGH ASYLUM.—Miss Peter, matron of Craig House, the private patient department of the Royal Edinburgh Asylum, was on Christmas Day presented by the nursing staff with a very handsome screen for her sitting-room.

LEIGH JOINT HOSPITAL.—On Christmas Day Miss Taylor, the matron of the Leigh Joint Hospital, was presented by the nurses with a pair of handsome silver mounted fish servers and silver gilt salts. On New Year's Day the servants presented the matron with a silver mounted crystal glass preserve dish and spoon, and the housekeeper with a pair of elegant silver gilt salts and spoons.

### Our Christmas Distribution.

WE have received the following letters, in addition to those already published, acknowledging parcels sent out:—

The MATRON of the Westminster Hospital writes:—I have much pleasure in thanking you for the very nice clothing you have been good enough to send for our patients. Not only will the garments give much pleasure to those who are fortunate enough to have them, but they are a great help to us as well, for it is such a comfort to have warm clothing to give away where it is needed.

THE MATRON of the North-Eastern Hospital for Children, Hackney Road, writes:—I beg to acknowledge the arrival of, and to thank you for, the nice Christmas gifts you have sent for the comfort and help of the children, when leaving the hospital, from the readers of THE HOSPITAL and the editors of THE HOSPITAL.

THE MATRON of the Children's Convalescent Home, Great Yarmouth, writes:—I beg to acknowledge with grateful thanks the parcel of useful clothing you have kindly sent for my children: they are most welcome, frocks especially.

### To Nurses.

WE invite contributions from any of our readers, and shall be glad to pay for "Notes on News from the Nursing World," or for articles describing nursing experiences, or dealing with any nursing question from an original point of view. The minimum payment for contributions is 5s., but we welcome interesting contributions of a column, or a page, in length. It may be added that notices of entertainments, presentations, and deaths are not paid for, but, of course, we are always glad to receive them. All rejected manuscripts are returned in due course, and all payments for manuscripts used are made as early as possible at the beginning of each quarter.

### Everybody's Opinion.

[Correspondence on all subjects is invited, but we cannot in any way be responsible for the opinions expressed by our correspondents. No communication can be entertained if the name and address of the correspondent is not given, as a guarantee of good faith but not necessarily for publication, or unless one side of the paper only is written on.]

### VISITING NURSES.

"B" writes: It does not seem to me that the work of the visiting nurse is clearly understood. The question of fees and time must be left to her discretion. She does whatever is necessary for the comfort of the patient, and leaves written instructions (when required) for what has to be done until her next visit. These instructions mainly consist of the diet, according to the doctor's directions, and the times specified for it, as well as the medicine to be given, which anybody with common sense can understand and carry out. It also falls to her lot occasionally to give a practical lesson in cooking—for the visiting nurse must understand cooking for the sick and convalescent. The floor and grate, &c., of the sick room are likewise attended to under her supervision. The circumstances of the case are always considered both in regard to time and charges, and though the visiting nurse has her card printed with certain rules, it is only done in self-defence against those people whose principles are uncultivated, and who would, if the time was not specified, expect the nurse's visit to last the greater part of the day, and also for those who plead that "they cannot afford to pay full fees" when they are able to surround themselves with every luxury that money can provide. Visiting nursing, from a pecuniary point of view, is by no means a desirable profession, on account of its uncertainty. Associations can, of course, be formed, and cheap labour can be procured, but no lasting good can ever come by sweating one lot of women to provide for the needs of another. Why not go to the root of the matter—to those who pay low wages for the services of educated ladies, and who are not ashamed of confessing that "they only employ them because they get them cheaper than men"? England is rich, and the public must learn to pay a fair wage for good services.

### RULES IN PRIVATE NURSING.

"THISTLE" writes: In all my cases where medicine has to be given four-hourly I inquire of the medical attendant if he wishes the patient to be roused for his medicine; if so, there is then, of course, no difficulty. Supposing the medicine has to be given four-hourly, and the patient is not to be disturbed if asleep—let us say it is due at two o'clock, and the patient does not awake within a quarter or half an hour, which brings us to half-past two—then I do not give the medicine until six o'clock. Some nurses, I know, object to this system, and give the medicine when the patient awakes, thus altering the hours of to-day from ten, two, and six to perhaps twelve, four, and eight, while to-morrow they may be eleven, three, and seven, or otherwise, on account of the patient having been asleep. I find the former plan suits better, when (as often happens) any of the friends have to relieve you for off-duty time. By having, as in the first case, stated hours, there is less danger of any mistake being made in her absence, especially if more than one drug has to be given. In the case of sleeping in a convalescent man's room I have no hesitation in writing "certainly not." I have been a private nurse for a little time now, but never has such an indignity been offered me. When the patient requires so little attention during the night, why not occupy a room, if possible, next his, and let him have a small hand-bell placed near his bed in case he really requires anything? If this is not convenient, then I would continue to sit with him during the night. Being so far convalescent one would have little difficulty to get the required amount of time off for sleep in the day. Like "A Watcher," I would almost rather lose the case than sleep in the same room, especially if the patient is so far convalescent as to be able to get up; and I see no reason why he requires the nurse's attendance during the night. In the last sentence of the letter I notice an allusion to young nurses. Why? Does "A Watcher" not think elderly nurses have just as much modesty as their younger sisters? By the tone of the letter I imagine she is a young nurse who has recently started private nursing. If this is so I would advise her to talk the subject of private nursing over with an elderly private nurse or, better, nurses. She will assuredly learn much.

### For Reading to the Sick.

"BE strong and of a good courage."

While many sympathising hearts
For my deliverance care,
Thou, in Thy wiser, stronger love,
Art teaching me to bear—
By the sweet voice of thankful song,
And calm, confiding prayer.

When I am feeble as a child,
And flesh and heart give way,
Then on Thy everlasting strength
With passive trust I stay;
And the rough wind becomes a song,
And darkness shines like day.

Oh, blessed are the eyes that see!
Though silent anguish show
The love, that in their hours of sleep
Unthanked may come and go;
And blessed are the ears that hear!
Though kept awake by woe,

There is no curse in this my pain,
For He was crucified;
And it is fellowship with Him
That keeps me near His side.—*A. L. Waring.*

Nay, all by Thee is ordered, chosen, planned—
Each drop that fills my daily cup; Thy hand
Prescribes for ills none else can understand.
All, all is known to Thee
—*A. L. Newton.*

If we stand in the openings of the present moment, with all the length and breadth of our faculties unselfishly adjusted to what it reveals, we are in the best condition to receive what God is always ready to communicate.
—*T. C. Upham*

God knows us through and through. Not the most secret thought, which we most hide from ourselves, is hidden from Him. As then we come to know ourselves through and through, we come to see ourselves more as God sees us, and then we catch some little glimpse of His designs with us, how each ordering of His Providence, each check to our desires, each failure of our hopes, is just fitted for us, and for something in our own spiritual state, which others know not of, and which, till then, we knew not. Until we come to this knowledge we must take all in faith, believing, though we know not, the goodness of God towards us. As we know ourselves, we, thus far, know God.—*E. B. Pusey.*

Nothing is intolerable that is necessary. Now God hath bound thy trouble upon thee, with a design to try thee, and with purposes to reward and crown thee. These cords thou canst not break; and therefore lie thou down gently, and suffer the hand of God to do what He please.—*Jeremy Taylor.*

I journey through a desert drear and wild,
Yet is my heart by such sweet thoughts beguiled,
Of Him on Whom I lean—my strength, my stay,
I can forget the sorrows of the way.

Thoughts of His love!—the root of every grace,
Which finds in this poor heart a dwelling-place;
The sunshine of my soul, than day more bright,
And my calm pillow of repose by night.

Thoughts of His coming!—for that joyful day
In patient hope I watch, and wait, and pray;
The dawn draws nigh, the midnight shadows flee,—
And what a sunrise will that advent be!

### Notes and Queries.

**The Editor is always willing to answer in this column, without any fee, all reasonable questions, as soon as possible.**
**But the following rules must be carefully observed:—**
**1. Every communication must be accompanied by the name and address of the writer.**
**2. The question must always bear upon nursing, directly or indirectly**
**If an answer is required by letter a fee of half-a-crown must be enclosed with the note containing the inquiry.**

*Meat Squeezer.*

(153) Will you kindly tell me where I can obtain the press for forcing juice from raw meat mentioned in Dr. Henderson's paper on beef juice?—*A. W.*

Messrs. R. and W. Wilson and Son, 90 Wardour Street, W.

*Visiting Nurses.*

(154) Will you kindly give me the name and address of the Secretary of the proposed Visiting Nurses' Organisation? I should be glad of any information respecting it.—*I. M. A.*

The scheme still seems in abeyance.

*Interrupted Career.*

(155) Would a certificated and experienced nurse be obliged to submit anew to training if she interrupted her nursing career by taking up other work, say for two or three years?—*C. D. R.*

Certainly not. It is frequently done.

*Baden Powell's Staff.*

(156) I wish to join Baden-Powell's staff. Will you kindly tell me to whom I should apply?—*Army Reserve Nurse.*

You can only apply to the ordinary authorities.

*Poro-plastic.*

(157) Can anyone tell me what to use to soften poro-plastic jacket?

The stiffening in poro plastic can be softened by heat. If it is desired to soften any particular part of a jacket this can be done either by the application of a hot sponge, or a hot water bottle, or if the jacket is not on the patient by holding a hot iron against it.

*Puerperal Fever.*

(158) Is puerperal fever always infectious and contagious? Would it be possible to nurse a case during the whole lying-in period in a ward with many recently delivered women without their becoming infected?—*Ignorant.*

A disease which is infectious does not always infect.

*Allowed.*

(169) Could you kindly tell me if a medical man is allowed to engage a midwife (L.O.S.) to attend his lying-in cases under his immediate personal supervision?—*C. H.*

Certainly, so long as he makes it clear that she is not employed by him to do work requiring professional discretion or skill, and that he is not "covering" her so as to enable her to attend and treat patients "as if the said person were duly qualified and registered." But she need not be an L.O.S. (a meaningless title, seeing that the letters L.O.S. stand for London Obstetrical Society), for anyone can attend women in labour. There is nothing to prevent a doctor turning a penny by any form of honest work. He may employ a servant to keep a shop, or he may part pay his groom's wages by letting him out to clean his neighbour's harness, or he may employ labourers to run a farm for him, and so long our lawmakers decide that no qualification is required for those who attend upon women in labour he may, if he likes, employ half a dozen charwomen to attend all the confinements in the village. So long as they are not "assisting" him or using his name to enable them to act as if they were "qualified and registered" under the Medical Act, he may invest his money in that as in any other enterprise And here comes in the oddity of the position; for, although so long as he merely employed women, trained or untrained, to attend upon confinements, and left them to play havoc with their cases, entirely unrestrained and unsupervised, he would break no law (any more than do the various lying-in charities which employ midwives for this work). Yet directly he tried to exercise some control over their performances, and to make them do their work carefully under his immediate personal supervision, that good doctor would be in immediate peril of being knocked off the register for knowingly allowing his assistant (for she would then be his assistant) to attend patients in respect of matters requiring professional discretion and skill, for it would be difficult, if not impossible, to draw the line between normal cases which the law relegates to the charwoman and cases which require treatment and professional skill at his hands. The law is indeed "a hass."

*Mucus.*

(160) Will you kindly tell me what mucus denotes in otherwise healthy urine, containing neither pus, blood, nor albumen? In what conditions of health or disease is it discovered?—*Nurse P. S.*

Mucus in the urine occurs wherever mucus is found in excess in any part of the urinary apparatus, and in women in many other conditions. The diagnosis of its cause is a somewhat complex business, quite outside the work of a nurse and too large a subject to enter on here.

### Standard Books of Reference.

"The Nursing Profession: How and Where to Train." 2s. net; post free, 2s. 4d.
"The Nurses' Dictionary of Medical Terms." 2s.
"Burdett's Series of Nursing Text-Books." 1s. each.
"A Handbook for Nurses." (Illustrated.) 5s.
"Nursing: Its Theory and Practice." New Edition. 3s. 6d.
"Helps in Sickness and to Health." Fifteenth Thousand.
"The Physiological Feeding of Infants." 1s.
"The Physiological Nursery Chart." 1s.; post free 1s. 3d.
"Hospital Expenditure: The Commissariat." 2s. 6d.

All these are published by THE SCIENTIFIC PRESS, Ltd., and may be obtained through any bookseller or direct from the publishers, 28 and 29 Southampton Street, London, W.C.

